# Relationship between interleukin (IL)-6 and brain morphology in drug-naïve, first-episode major depressive disorder using surface-based morphometry

**DOI:** 10.1038/s41598-018-28300-5

**Published:** 2018-07-03

**Authors:** Shingo Kakeda, Keita Watanabe, Asuka Katsuki, Koichiro Sugimoto, Natsuki Igata, Issei Ueda, Ryohei Igata, Osamu Abe, Reiji Yoshimura, Yukunori Korogi

**Affiliations:** 1 Department of Radiology, University of Occupational and Environmental Health, Kitakyushu, Japan; 2 Department of Psychiatry, University of Occupational and Environmental Health, Kitakyushu, Japan; 3 Department of Radiology, Graduate School of Medicine, the University of Tokyo, Tokyo, Japan

## Abstract

There is a growing body of evidence to support the involvement of proinflammatory cytokines in the pathophysiology of depression; however, no previous studies have examined the relationship between cytokines and the brain morphology of patients with major depressive disorder (MDD). We therefore evaluated the relationship between serum cytokine levels and cortical thinning during the first depressive episode in drug-naïve patients with MDD. We measured the serum cytokine levels (IL-1β, IL-6, IFN-γ, and TNFα), and whole-brain cortical thickness and hippocampal subfield volumes on brain magnetic resonance imaging (MRI) using surface-based morphometry in 40 patients with MDD and 47 healthy volunteers (controls). Only the serum IL-6 level was significantly higher in patients with MDD than in controls. The prefrontal cortex (PFC) thickness was significantly reduced in patients with MDD, and showed a significant inverse correlation with the serum IL-6 level. Although high serum IL-6 levels were correlated with reduced left subiculum and right CA1, CA3, CA4, GC-DG, subiculum, and whole hippocampus volumes, the presence or absence of MDD had no effect on the volume of any hippocampal subfields. Our results suggest that IL-6 may play a key role in the morphological changes in the PFC during the early stage of MDD.

## Introduction

Although there have many theories proposed regarding the cause of major depressive disorder (MDD), the pathogenesis is only partly understood; genes, the environment, and endocrine dysfunction are all considered to be factors influencing MDD^1^. Accumulating evidence suggests a role of inflammation in the pathogenesis of MDD. For example, several studies have detected higher levels of inflammation in MDD than in healthy controls; however, the strength of the evidence varies according to the specific inflammatory markers that have been examined (*i.e*., interleukin [IL]-1β, IL-6, interferon γ [IFN-γ], and tumor necrosis factor α [TNFα])^2–4^.

In several animal models, increased levels of serum proinflammatory cytokines, such as IL-6, were shown to interfere with long-term potentiation^5,6^, neurogenesis^7^, and neural plasticity^8^. Moreover, transgenic mice with high IL-6 expression levels showed neuropathologic manifestations, including neurodegeneration^9^. This evidence suggests that cytokines may affect the brain morphology through processes related to neurodegeneration. To the best of our knowledge, only one previous study has evaluated the relationship between brain morphology and the serum cytokine levels in patients with MDD; the results showed that the increased expression of IL-6 predicted a decreased hippocampal volume^10^. However, the authors of that study only evaluated the hippocampal and amygdala structures using a manual tracing method. Furthermore, the study was limited by the fact that two-thirds of patients were on antidepressant medications—according to a meta-analysis^11^, treatment with antidepressants reduces the serum IL-6 levels in patients with MDD.

Many investigators have applied PET to examine a marker of neuroinflammation, translocator protein (TSPO) binding, *in vivo*, to test the neuroinflammatory hypothesis of MDD^12–15^. Several studies have shown that—in addition to the hippocampus—neuroinflammation was present in various brain regions of patients with MDD^12–14^. This evidence led us to wonder whether elevated cytokine levels might be associated with a reduced cortical volume not only of the hippocampus, but also extend into other brain regions in patients with MDD. To test this possibility, we employed a surface-based morphology (SBM) analysis, which has been proposed to identify the differences in the thickness of gray matter on the brain’s surface^16^, as a whole-brain voxel based-morphometry (VBM) analysis procedure. We also added a recently developed hippocampal subfield analysis using the automated hippocampal subfield segmentation method^17^, because it is not possible to analyze the hippocampus by a conventional SBM analysis. This technique allows for the calculation of the hippocampal subfield volumes. The purpose of the current study was to investigate the relationship between the brain morphology (brain cortical thinning and hippocampal subfield volumes) and the serum cytokine levels during the first depressive episode in drug-naïve patients with MDD using a whole-brain SBM analysis.

## Materials and Methods

### Participants

Human experiments were carried out in accordance with guidelines provided and approved by the Institutional Review Board of University of Occupational and Environmental Health School of Medicine, Japan (approval number: H25-13). The protocol of this prospective study was approved by the Ethics Committee of the University of Occupational and Environmental Health. All of the participants provided their written informed consent to participate in the study.

In the current study, first-episode and drug-naïve patients with MDD were recruited. A psychiatrist (A.K., with 12 years of experience in psychiatry) diagnosed patients with MDD using a fully Structured Clinical Interview for Diagnostic and Statistical Manual for Mental Disorders, Fourth Edition, Text revision (DSM-IV-TR) Research Version, Non-Patient Edition (SCID-I/NP). To qualify for the study, patients with MDD must not have previously met the criteria for any DSM-IV-TR Axis I disorder during interviews performed by a psychiatrist. In short, none of the patients with MDD in this study had any past episodes of mood disorders. Moreover, patients with mild cognitive impairment were excluded mainly based on information about their activities of daily living from family members or caregivers, in addition a brief cognitive examination including a serial 7 s test and an assessment of the patient’s short-term memory was performed by an experienced psychiatrist.

The severity of depression was evaluated using the 17-item Hamilton Rating Scale for Depression (HAMD-17). Only patients with a total HAMD-17 score of ≥14 were eligible for inclusion in the study. Between March 2009 and January 2017, 50 consecutive patients were recruited. From this initial sample, the psychiatrist excluded patients who met the following criteria: (a) a history of neurological disease or the presence of either Axis I (schizophrenia, other affective disorders, etc.) or Axis II (personality disorders, mental retardation, etc.) psychiatric disorders (n = 5); (b) presence of co-morbid substance use disorders (n = 3); (c) unwillingness to provide informed consent (n = 2). Thus, a total of 40 right-handed, first-episode, drug-naïve patients with MDD were included (Table 1). Thirty of the 40 patients had participated in our previously published studies^18,19^, which analyzed the relationship between brain structures and the serum cortisol levels in MDD.

**Table 1:** The demographic characteristics, cytokine values, and brain volumes of participants.

	Healthy subjects (n = 47)	MDD patients (n = 40)	p-value
Age, mean, (range, SD)	40.7 (20-65, 11.4)	46.6 (20–67, 14.4)	0.06
Female, numbers	13	20	0.05
Obesity, %	3 (6.4)	2 (5.0)	1.00
HAMD-17, mean of total scores (SD)		21.6 (5.9)	
Duration of depressive episode, month (SD)		6.3 (10.2)	
ICV, mean (SD)	1628.2 (160.6) ml	1551.8 (145.3) ml	0.58
**Inflammatory, mean; pg/ml (SD)**			
IL-1β	0.053 (0.087)	0.041 (0.048)	0.23
IL-6	0.370 (0.327)	0.850 (1.808)	0.04
IFN-γ	7.459 (13.4)	9.636 (15.0)	0.24
TNF-α	2.041 (5.39)	1.592 (0.576)	0.30

SD, standard deviation; MDD, Major depression disorder; HAMD, 17-item Hamilton Rating Scale for Depression; ICV, intracranial volume; IL, interleukin; IFN, interferon; TNF, tumor necrosis factor. Obesity was defined by body mass index (>26 kg/m^2^).

Fifty-seven healthy volunteers (controls) were also recruited from nearby communities via an interview conducted by the same psychiatrist using the full SCID-I/NP. None of the control participants had a history of serious medical or neuropsychiatric illness or a family history of major psychiatric or neurological illness among their first-degree relatives (Table 1).

A radiologist (S.K., 21 years of experience in neuroradiology) who reviewed the conventional magnetic resonance imaging (MRI) data (including T2-weighted images) reported no gross abnormalities such as infarcts, hemorrhages, or brain tumors in any of the study participants.

### Cytokine analysis

Forty human blood samples were assayed in singlicate (due to limited sample volumes) using the V-PLEX Human Proinflammatory Panel I (4-Plex), a highly sensitive multiplex enzyme-linked immunosorbent assay used to quantitatively measure cytokines, including IL-1β, IL-6, IFN-γ, and TNFα, from a single small sample volume (25 μL) using an electrochemiluminescent detection method (MesoScale Discovery, Gaithersburg, MD, USA) (Table 1). The mean intra-assay coefficients, based on the standards run in duplicate for each cytokine, were <8.5% for all cytokines. For the purposes of the statistical analysis, any value that was below the lowest limit of detection (LLOD) for the cytokine assay was replaced with half of the LLOD of the assay. This imputation method is robust and well established^20^.

### MRI acquisition

MRI was performed using a 3T MR system (Signa EXCITE 3 T; GE Healthcare, Wankesha, WI, USA) with an 8-channel brain phased-array coil. Original T_1_ images were acquired by three-dimensional fast-spoiled gradient recalled acquisition with steady state. The acquisition parameters were as follows: repetition time, 10 ms; echo time, 4.1 ms; inversion time, 700 ms; flip angle, 10; field-of view, 24 cm; section thickness, 1.2 mm; and resolution, 0.9 × 0.9 × 1.2 mm. All images were corrected for image distortion due to gradient non-linearity using the Grad Warp software program^21^ and for intensity inhomogeneity with the “N3” function^22^.

### Image processing

#### Whole-brain analyses using SBM

The regional cortical thickness was estimated using the FreeSurfer software program (version 6.0, www.freesurfer.net/fswiki/HippocampalSubfields), which has been well documented and which is freely available online. The technical details of the cortical thickness analysis have been described elsewhere^23^. The entire cortex of each participant was inspected visually; topological defects were corrected manually. Cortical thickness measurements were obtained by reconstructing representations of the gray matter–white matter boundary^23,24^ and the pial surface. The distance between these surfaces at each point across the cortical mantle was then calculated. For each participant, the regional thickness value at each vertex was mapped to the surface of an average brain template. This allowed for the visualization of data across the entire cortical surface. The data were re-sampled for all participants onto a common spherical coordinate system^24^. The cortical map of each participant was smoothed with a 10-mm kernel in full width at half-maximum (FWHM) for the cortical analyses.

### Volumetry of the hippocampal subfields

**Figure 1:**
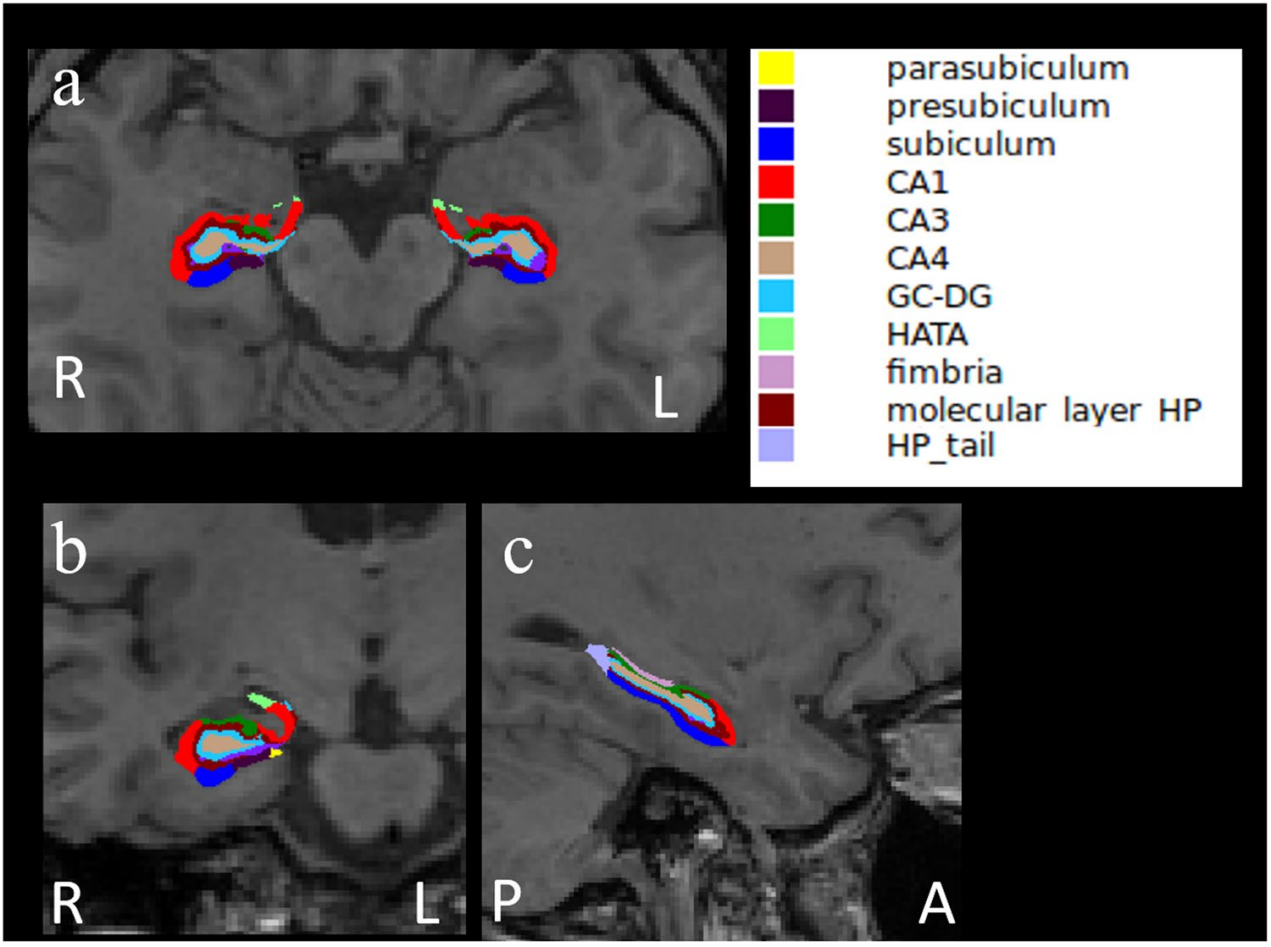
The representative subdivision of the hippocampal subfields. The mask of each region overlapped on the axial (**a**), coronal (**b**), and sagittal (**c**) T1-weighted images. R, right; L, left; A, anterior; P, posterior. Color classification: parasubiculum, yellow; presubiculum, dark purple; subiculum, blue; CA1, red; CA3, dark green; CA4, brown; granule cell layer of dentate gyrus (GC-DG), sky blue; hippocampus-amygdala-transition-area (HATA), green; fimbria, purple; molecular layer hippocampus (HP), dark brown; hippocampal tail, gray.

The FreeSurfer software program (version 6.0 www.freesurfer.net/fswiki/ HippocampalSubfields)^25^ was used to calculate hippocampal subfield volumes. The hippocampal subfields were segmented using a Bayesian inference approach and a novel atlas algorithm of the hippocampal formations built primarily upon ultra-high resolution *ex vivo* MRI data from autopsy brains^26^. This presented atlas was more sensitive than the previous version (FreeSurfer 5.3) that used as ab *in vivo* atlas for segmenting the hippocampal subfields as it allowed for greater accuracy in the delineation of the boundaries within the subfields^26^. The calculated sub-regions included Cornu Ammonis (CA)1, CA3, CA4, the granule cell (GC) layer of the dentate gyrus (DG) (GC-DG), fimbria, subiculum, presubiculum, parasubiculum, molecular layer, hippocampus-amygdala-transition-area, hippocampal tail, and whole hippocampus (Fig. 1). We calculated the total hippocampal volume in each hemisphere as the sum of the volumes of all subfields except the hippocampal fissure. The technical details of these procedures have been described elsewhere^26^.

### Statistical analyses

As age and the serum cytokine levels exhibited Gaussian distributions, we applied independent sample *t*-tests to assess the differences between healthy participants (“controls”) and patients with MDD (“patients”). The chi-squared test was used for gender comparisons.

To investigate differences in the cortical thickness of patients and controls and to assess the relationship between cortical thickness and serum cytokine levels, we performed SBM using the FreeSurfer QDEC statistical tool after 10-mm FWHM kernel smoothing. A general linear model was then applied at each vertex. The following comparisons were performed in a whole-brain vertex-by-vertex analysis: (a) comparison between controls and patients; (b) correlation between cortical thickness of patients and serum cytokine levels; and (c) correlation between cortical thickness of controls and serum cytokine levels. We set the diagnosis as “discrete” and serum cytokine levels as “continuous.” In addition, age and gender were set as “nuisance factors” to control for confounding variables. It was plausible that the controls and patients included in this study would show different cortical evolution rates; thus, different offsets, different slopes (known as “DODS”) was employed. To correct for multiple comparisons, we used a Monte Carlo simulation for the cluster analysis. The cluster-forming threshold was set at *p* < 0.05. Clusters were then tested against an empirical null distribution of maximum cluster size built using synthesized Z-distributed data across 10,000 permutations, producing cluster-wise *p*-values that were fully corrected for multiple comparisons.

We performed a multiple linear regression analysis to evaluate the relationship between the effects of the diagnosis (MDD vs. control) and the hippocampal subfield volumes. A multiple linear regression model was also used to evaluate whether serum proinflammatory cytokines were related to the hippocampal subfield volumes. Age, gender, and intracranial volume were entered as covariates in both analyses.

*P* values of <0.05 were considered to indicate statistical significance. The statistical analyses were performed using the R software program (version 3.4.0, R Statistical and Computing Software; http://www.r-project.org/).

## Results

### Baseline demographic data

Table 1 shows participants’ baseline demographic data. There were no significant differences in age, gender, or the presence of obesity between controls and patients. The serum IL-6 levels of the patients were significantly higher in comparison to controls.

None of the serum cytokine levels were associated with the total HAMD-17 score or the duration of the depressive episode in the MDD patients (by Spearman’s rank correlation). The serum cortisol levels were measured in 32 of the 40 MDD patients. Although a previous study showed associations between cytokine levels and the serum cortisol level^27^, we found no significant correlation between the serum cortisol level and the levels of various cytokines, including IL-6 (IL-6; *p* = 0.78 by Spearman rank correlation).

### Whole-brain analyses using SBM

**Figure 2:**
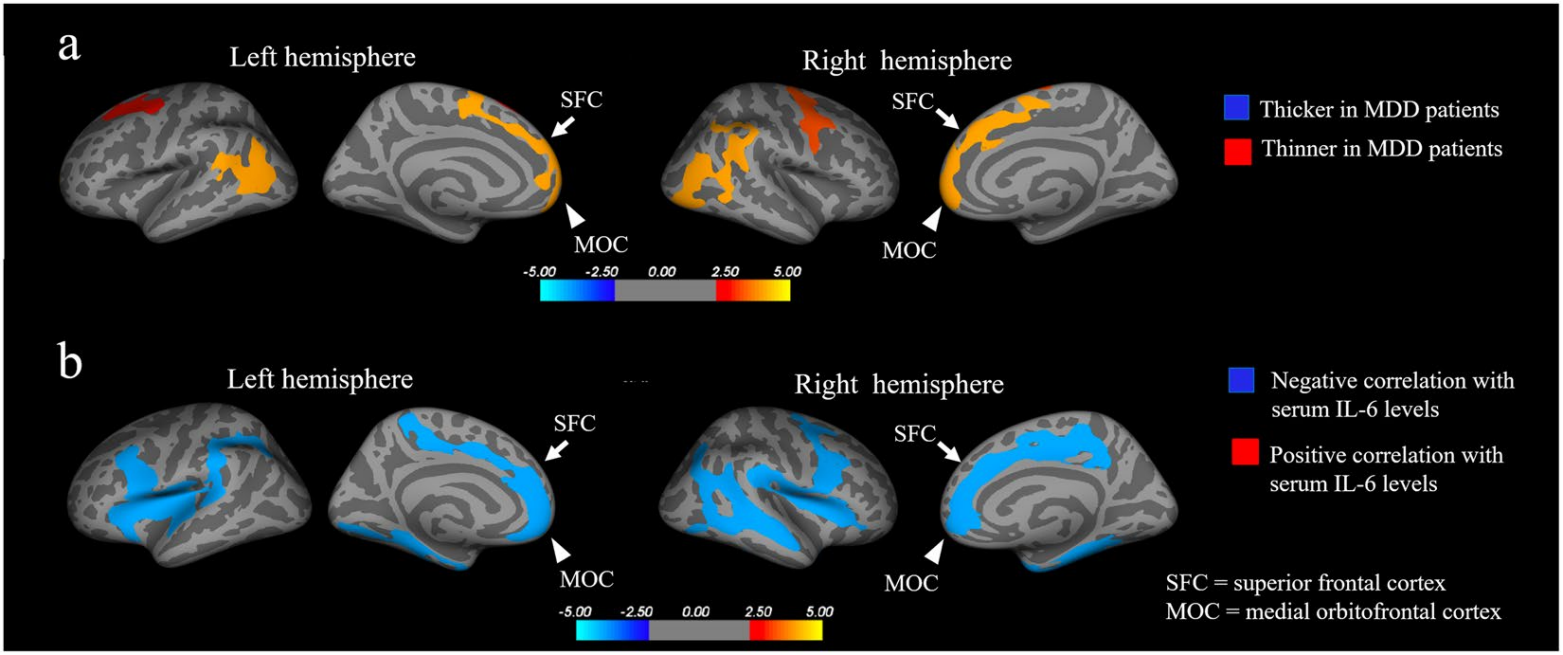
The whole-brain analysis using surface-based morphology (SBM). (**a**) Comparison of the cortical thickness in MDD patients vs. healthy controls. Orange or red clusters represent the significantly thinner cortical regions in patients with MDD (*p* < 0.05 vs. controls, Monte Carlo simulation). The thickness of the bilateral superior frontal (arrows) and medial orbitofrontal (arrow heads) cortices of patients with MDD was significantly lower in comparison to controls (*p* < 0.05, Monte Carlo simulation). **(b)** The relationship between the cortical thickness and the serum IL-6 levels in patients with MDD. Blue clusters represent regions in which a significant negative correlation was between the cortical thickness and serum IL-6 levels in patients with MDD (*p* < 0.05, Monte Carlo simulation). The thickness of the bilateral superior frontal (arrows) and medial orbitofrontal (arrow heads) cortices exhibited a significant negative correlation with the serum IL-6 level (*p* < 0.05, Monte Carlo simulation).

The coordinates of the significantly thinner cortical regions in patients are presented in Table 2. In group comparisons using a whole-brain vertex-by-vertex analysis adjusted for age and gender, the thickness of the bilateral superior frontal and medial orbitofrontal cortices of patients were significantly lower (*p* < 0.05, Monte Carlo simulation) (Fig. 2a; orange or red clusters). There were no regions in which the cortical thickness of patients was significantly greater in comparison to controls.

**Table 2:** Whole brain analyses using surface-based morphometry.

Cortical regions	Max	VtxMax	Size (mm^2^)	TalX^b^	TalY^b^	TalZ^b^	CWP
Thinner in MDD patients^a^							
Left hemisphere							
inferior parietal and lateral occipital cortices	4.051	27613	2026.93	−40.1	−62.0	6.6	0.0004
superior frontal, medial orbitofrontal cortices	2.640	152852	1948.08	−8.2	2.2	50.3	0.0013
caudal middle frontal cortices	3.084	105971	1511.15	−27.5	5.8	46.4	0.0095
Right hemisphere							
superior frontal and medial orbitofrontal cortices	3.002	92871	1978.74	8.0	19.7	41.2	0.0012
precentral cortex	3.376	140561	1929.42	50.4	1.2	36.7	0.0013
inferior parietal and supra-marginal cortices	3.814	33905	1868.01	52.9	−45.0	27.2	0.0015
lateral occipital cortex	4.195	158509	1508.89	47.1	−68.8	12.4	0.0089
Correlation with serum IL-6 level in MDD patients^a^							
Left hemisphere							
triangularis	−4.309	16892	8536.62	−29.5	22.1	9.3	0.0001
superior frontal, medial orbitofrontal, and paracentral cortices	−3.701	64159	3064.38	−13.5	44.6	1.0	0.0001
parahippocampal cortex	−4.928	70540	2386.87	−28.1	−30.6	−14.5	0.0001
rostral middle frontal cortex	−2.691	149979	1498.34	−22.1	38.7	19.7	0.0168
middle temporal cortex	−4.576	24712	1421.53	−47.2	−33.3	−4.9	0.0218
Right hemisphere							
caudal middle frontal cortex, insula, and opercularis	−4.132	23947	5844.73	31.1	17.1	9.8	0.0001
superior and middle temporal cortices	−4.777	163449	5260.70	47.2	−21.7	−10.2	0.0001
superior frontal, medial orbitofrontal, and cingulate cortices	−4.888	12712	3690.45	13.6	−40.6	32.6	0.0001
parahippocampal cortex	−4.470	9976	2465.30	32.1	−29.5	−13.9	0.0001

MDD, major depressive disorder; CWP = opercularis clusterwise p value.

^a^Significantly thinner cortical regions and IL-6-associated regions in MDD patients were detected using FreeSurfer v 6.0. p < 0.05 (Monte-Carlo simulation).

^b^Based on Talairach and Toumoux system.

The coordinates of the cortical regions, which showed a significant correlation with the serum cytokine levels, are presented in Table 2. A whole-brain vertex-by-vertex correlation analysis of the patients showed that the thickness of the bilateral superior frontal and medial orbitofrontal cortices was significantly negatively correlated with the serum IL-6 level (*p* < 0.05, Monte Carlo simulation) (Fig. 2b; blue clusters). There were no regions manifesting a significant positive correlation in patients. Furthermore, no brain regions showed significant correlations with the levels of other cytokines (IL-1β, IFN-γ, and TNFα). Finally, we found no regions in which the cortical thickness was significantly correlated with the serum cytokine levels in controls.

There were no brain regions in which the cortical thickness was associated with the total HAMD-17 score or the duration of the depressive episode.

### Volumetry of the hippocampal subfields

In multiple linear regression models adjusted for age, gender, and intracranial volume, high serum IL-6 levels were significantly negatively correlated with reduced left subiculum and right CA1, CA 3, CA 4, GC-DG, subiculum, and whole hippocampus volumes (Table 3). However, we found no effects of the diagnosis (patient vs. control) on the volume of any hippocampal subfields.

**Table 3:** The relationships among hippocampal subfield volumes and serum proinflammatory cytokines.

Hippocampal subfields	IL-1β			IL-6			IFN-γ			TNF-α			Effects of diagnosis (MDD vs. control)		
	B	SE	p	B	SE	p	B	SE	p	B	SE	p	B	SE	p
Left															
CA1	−0.108	0.164	0.515	−0.32	0.167	0.064	0.097	0.164	0.56	0.119	0.183	0.518	<0.001	0.115	0.998
CA3	−0.128	0.167	0.45	−0.244	0.172	0.164	0.088	0.166	0.598	−0.08	0.185	0.67	−0.013	0.116	0.911
CA4	−0.071	0.161	0.66	−0.262	0.164	0.119	0.082	0.159	0.609	0.04	0.178	0.823	−0.009	0.115	0.941
GC-DG	−0.079	0.156	0.618	−0.268	0.159	0.1	0.086	0.155	0.581	0.064	0.173	0.713	−0.071	0.113	0.535
fimbria	0.087	0.145	0.556	0.169	0.149	0.262	0.221	0.137	0.116	0.139	0.157	0.381	−0.163	0.105	0.125
subiculum	0.171	0.159	0.291	−0.339	0.161	0.042*	0.11	0.159	0.494	0.189	0.176	0.292	−0.038	0.112	0.734
presubiculum	0.066	0.162	0.687	−0.106	0.167	0.529	−0.191	0.155	0.225	0.167	0.175	0.346	−0.178	0.113	0.118
parasubiculum	0.154	0.171	0.376	−0.038	0.181	0.833	−0.294	0.163	0.08	0.052	0.19	0.788	−0.116	0.115	0.319
molecular layer	<0.001	0.156	0.998	−0.253	0.16	0.122	0.015	0.156	0.923	0.102	0.173	0.562	−0.054	0.113	0.635
HATA	−0.062	0.167	0.714	0.033	0.174	0.852	−0.011	0.163	0.947	−0.212	0.179	0.244	−0.2	0.112	0.079
hippocampal tail	0.011	0.157	0.947	−0.186	0.161	0.254	−0.041	0.154	0.789	0.109	0.171	0.528	0.029	0.11	0.794
whole hippocampus	0.006	0.153	0.97	−0.288	0.155	0.071	0.03	0.152	0.843	0.136	0.169	0.426	−0.059	0.113	0.599
Right															
CA1	−0.026	0.162	0.871	−0.347	0.162	0.039*	0.098	0.161	0.549	0.054	0.181	0.765	0.058	0.114	0.612
CA3	−0.081	0.159	0.613	−0.329	0.158	0.045*	−0.032	0.157	0.841	−0.127	0.174	0.471	0.139	0.113	0.223
CA4	0.057	0.156	0.717	−0.323	0.156	0.046*	0.006	0.155	0.968	−0.046	0.173	0.793	0.042	0.114	0.712
GC-DG	0.051	0.154	0.744	−0.355	0.152	0.025*	0.012	0.153	0.94	−0.036	0.171	0.832	−0.008	0.113	0.943
fimbria	−0.031	0.155	0.842	0.149	0.158	0.35	0.073	0.15	0.627	−0.134	0.166	0.425	−0.114	0.103	0.272
subiculum	0.11	0.171	0.525	−0.261	0.175	0.144	−0.056	0.169	0.741	0.152	0.187	0.422	−0.048	0.115	0.674
presubiculum	0.106	0.167	0.53	−0.034	0.175	0.849	−0.289	0.157	0.075	0.074	0.184	0.69	−0.174	0.113	0.129
parasubiculum	0.073	0.166	0.664	0.073	0.175	0.677	0.072	0.164	0.663	−0.072	0.184	0.699	−0.025	0.115	0.826
molecular layer	0.022	0.161	0.891	−0.297	0.162	0.075	−0.012	0.159	0.942	0.024	0.178	0.893	−0.019	0.113	0.87
HATA	−0.006	0.156	0.968	−0.092	0.166	0.583	0.178	0.153	0.253	−0.116	0.174	0.509	−0.072	0.114	0.526
hippocampal tail	0.097	0.154	0.534	−0.332	0.152	0.036*	−0.031	0.152	0.84	0.142	0.169	0.407	−0.12	0.104	0.252
whole hippocampus	0.051	0.156	0.746	−0.326	0.155	0.043*	−0.017	0.155	0.912	0.045	0.173	0.797	−0.042	0.111	0.705

B (SE) 5 b value (standard error of the mean). MDD = Major depression disorders, IL = Interleukin, IFN = interferon, TNF = Tumor necrosis factor. CA = cornu ammonis, GC-DG = granule cell layer of dentate gyrus, HATA = hippocampus-amygdala-transition-area, hippocampal tail, and whole hippocampus. Age, sex, and intracranial volume are entered as covariates in analyses. *Significant correlation between the hippocampal subfield volumes and serum cytokine levels in MDD patients are detected (p < 0.05).

## Discussion

A strength of this study lies in recruitment of first depressive episode and drug-naïve patients with MDD. To our knowledge, this is the first study to investigate which brain region is related to serum cytokines levels by using the whole-brain SBM analysis. We found that the cortical thicknesses in most regions of the superior frontal and medial orbitofrontal cortices were significantly negatively correlated with the serum IL-6 level. Moreover, the thickness of the superior frontal and medial orbitofrontal cortices in MDD patients was significantly decreased in comparison to healthy controls. Thus, our results suggest that the neuroinflammatory status in the early stage of MDD is associated with changes in the gray matter.

The serum IL-6 level was significantly higher in patients with MDD than in controls; this was the only cytokine among the cytokines that we tested that differed between patients and controls. A previous meta-analysis demonstrated that the concentrations of the serum TNF-α and IL-6 levels in patients with MDD were significantly higher in comparison controls^2^. A more recent meta-analysis also demonstrated that the cerebrospinal fluid levels of IL-6 in patients with MDD were higher than those in controls^28^. Finally, the third meta-analysis demonstrated that treatment with antidepressants reduced the serum IL-6 levels in patients with MDD^11^. Thus, the presence of increased circulating concentrations of IL-6 in MDD is well established and was confirmed in the current study.

IL-6 is a multifunctional cytokine that regulates the growth and differentiation of various tissues, and which plays an important role in the immune response and acute-phase reactions^29^. IL-6 has been proposed to be involved in the pathology of MDD^30^. In particular, it has been suggested that IL-6 is involved in multiple physiological systems, including the hypothalamic-pituitary-adrenal axis, corticotrophin-releasing hormone activity at limbic sites, noradrenaline utilization, the induction of oxidative stress, apoptotic pathways, and kinase signaling^31–34^, all of which have very close relationships with the pathophysiology of MDD. Moreover, a previous study demonstrated that IL-6 directly controlled the serotonin transporter (SERT) level and consequently serotonin reuptake^35^. The SERT activity shapes serotonergic neurotransmission, which is implicated in the behavioral features and pathophysiology of MDD^36^. More recently, Igata *et al*. reported that the serotonin transporter genotype was associated with the volume of the gray matter in MDD patients^37^. Thus, further studies are required to explain the physiological systems in which IL-6 might be related to gray matter changes.

Most superior frontal and medial orbitofrontal cortices, which were determined in this study, are occupied by the PFC. The PFC has been implicated in the mediation of emotional and autonomic responses to socially significant or provocative stimuli, and specific PFC abnormalities have been implicated in the pathophysiology of mood disorders, including MDD^38,39^. Many previous neuroimaging studies have suggested that PFC abnormalities are important in the pathophysiology of MDD. Salvadore *et al*. showed a reduction in the volume of the PFC by a VBM analysis^40^, and Koolschijn *et al*.^41^ and Bora *et al*.^42^ conducted meta-analyses of VBM studies and demonstrated a reduced PFC volume in patients with MDD. A number of positron emission tomography studies have repeatedly identified a decrease in the PFC metabolic activity in patients with MDD^43–45^. Our findings are consistent with these previous studies, and also indicate that alterations of the PFC are already present in the early stage of MDD.

Setiawan *et al*. detected inflammation in the brains of patients with MDD, as indicated by increased TSPO VT, and this inflammation was prominent in the PFC, anterior cingulate cortex, and insula^14^. These results indicate that the PFC seems to be more sensitive and vulnerable to increased inflammatory conditions in comparison to other brain regions. This hypothesis may also be supported by previous studies which showed that IL-6 receptors were concentrated in the PFC^46–48^.

To our knowledge, this is the first study to investigate the influence of proinflammatory cytokines on the hippocampal subfield volumes in MDD patients. We found that high serum IL-6 levels were correlated with reduced volumes of the CA1-3, subiculum, and DG. A hippocampal subfield volume analysis using 4.7 T MRI showed that MDD patients had a lower CA1-3 and DG volumes in comparison to healthy controls^49^. Lim *et al*. who also used the same method as our study, reported that patients with late-life depression show reduced CA 2–3 volumes in comparison to healthy controls^50^. Several animal studies have reported a massive neuronal loss of CA3 pyramidal cells, dendritic retraction in CA1-3 and the DG, and suppressed DG neurogenesis and glial loss in the hippocampi of severely stressed animals^51–53^. These studies may support our results because acute or chronic stress led to increased proinflammatory cytokine production^54^. However, in the present study, there were no significant differences in any of the hippocampal subfield volumes between patients and controls. This finding may suggest that the hippocampal subfield volumes may be maintained in the early stage of first-episode MDD, and the volume reduction may occur after the reduction of the PFC volumes. Another explanation for our negative result may be that the pathological changes in the volumes of the hippocampal subfields may be too subtle to detect by 3 T MRI.

Our study was associated with some limitations. First, the study population was relatively small and all of the patients recruited were recruited from a single institution; thus, there is a risk of sampling bias. However, it was difficult to recruit and retain drug-naïve patients with MDD during their first episode because many patients received antidepressants before they underwent MRI. Second, we did not evaluate environmental stress in the present study. Environmental stress is a major trigger of proinflammatory cytokine production^51–54^ and affects the brain morphology^55,56^. Further studies on the relationship between environmental stress, the symptoms and behaviors of patients with MDD, and investigations of gray matter changes are needed. Furthermore, we did not perform cognitive tests such as the Mini-Mental State Examination (MMSE). Thus, we cannot rule out the possibility that cognitive functions affected the results. Moreover, the white matter abnormalities constitute one element of the pathogenesis of MDD. Thus, using diffusion tensor imaging (DTI), investigations of the relationship between the integrity of pathways within relevant neural networks and the serum cytokine levels are currently underway in our laboratory. Third, we focused on a cross-sectional association based on one-time assessments of inflammatory marker levels, although these measurements cannot reliably distinguish between chronic and acute inflammation. The few previous studies that have assessed chronic inflammation have revealed stronger associations with mental health when inflammation is determined using repeated measurements rather than a single measurement^57^. Fourth, we only evaluated the first depressive episode in drug-naïve patients with MDD. Thus, longitudinal work with a larger sample of patients with MDD under various conditions will be required to determine causal links between the serum IL-6 level and alterations in brain morphology. Finally, the difference in the age of the patients and controls was almost statistically significant (p = 0.06), which might have affected the results. Age-related chronic inflammation and dysregulated immune activation are considered to be mechanisms of immunosenescence. Actually, a previous study of a geriatric population showed that serum IL-6 elevation was associated with aging^58^.

In conclusion, we found that the serum IL-6 levels of patients with MDD were significantly higher in comparison to controls. Importantly, the PFC thickness of patients with MDD was significantly reduced, and showed a significant inverse correlation with the serum IL-6 level. Since the PFC contains a high concentration of IL-6 receptors, IL-6 receptor-mediated neurotoxicity might occur under the high serum IL-6 levels that are present in the early stage of MDD. Our results suggest that IL-6 may play a key role in the changes in brain morphology that occur in the early stage of MDD.
